# Some novel mathematical analysis on the fractional‐order 2019‐nCoV dynamical model

**DOI:** 10.1002/mma.8772

**Published:** 2022-10-04

**Authors:** Abiodun Ezekiel Owoyemi, Ibrahim Mohammed Sulaiman, Pushpendra Kumar, Venkatesan Govindaraj, Mustafa Mamat

**Affiliations:** ^1^ Department of General Studies Federal College of Agricultural Produce Technology Kano Nigeria; ^2^ Institute of Strategic Industrial Decision Modelling (ISIDM), School of Quantitative Sciences Universiti Utara Malaysia Sintok 06010 Kedah Malaysia; ^3^ Institute for the Future of Knowledge University of Johannesburg PO Box 524 Auckland Park 2006 South Africa; ^4^ Department of Mathematics National Institute of Technology Puducherry Karaikal 609609 India; ^5^ Faculty of Informatics and Computing Universiti Sultan Zainal Abidin Kuala Terengganu Malaysia

**Keywords:** Caputo derivative, equilibrium points, fractional mathematical model, stability analysis

## Abstract

Since December 2019, the whole world has been facing the big challenge of Covid‐19 or 2019‐nCoV. Some nations have controlled or are controlling the spread of this virus strongly, but some countries are in big trouble because of their poor control strategies. Nowadays, mathematical models are very effective tools to simulate outbreaks of this virus. In this research, we analyze a fractional‐order model of Covid‐19 in terms of the Caputo fractional derivative. First, we generalize an integer‐order model to a fractional sense, and then, we check the stability of equilibrium points. To check the dynamics of Covid‐19, we plot several graphs on the time scale of daily and monthly cases. The main goal of this content is to show the effectiveness of fractional‐order models as compared to integer‐order dynamics.

## 1 INTRODUCTION

In recent times, Covid‐19/2019‐nCoV/Coronavirus was classified as a deadly epidemic for humans. Lots of families have lost their relatives affected by the disease. With the discovery of many variants of Covid‐19, we can say that the disease is not yet over. Until this moment, many nations are still reporting new cases and deaths of individuals infected by the virus. The efforts from several research units to present vaccines against the spread of this virus to protect the population have been influenced by the discoveries of new variants. Researchers suggest that vaccines are slightly less effective against most of the variants and, thus, the variance can spread freely. As we believe that for any disease, when we don't have a complete treatment, and then, mathematical models always become useful to predict the behavior of the epidemic in the future.

Currently, different types of mathematical models have been used to study the dynamics of Covid‐19 in which classical and non‐classical (fractional‐order) models have been justified. Since the discovery of this virus, a very large wave of applications of fractional derivatives has been introduced in different scientific fields. Particularly, a number of studies related to Covid‐19 fractional‐order modelings have been given by many researchers. Gao et al^1^ have simulated the dynamics of early unreported cases of Covid‐19. In Erturk and Kumar,^2^ a new generalized Caputo‐type fractional derivative has been used to simulate the dynamics of a Coronavirus model.^3^ In Nabi et al,^4^ the structure of Covid‐19 disease in Cameroon has been investigated. In Kumar and Suat Erturk,^5^ a solution of a time‐delay fractional‐order Covid‐19 model is also given. Projections of the Covid‐19 data along with optimal control strategies are given in Nabi et al.^6^ Predictions on the epidemic peaks of Covid‐19 in Brazil are proposed in Kumar et al^8^ have proposed a novel study on Covid‐19 by analyzing the role of the vaccine. Kumar and Suat Erturk^9^ simulated the 2019‐nCoV cases in India via fractional derivatives.

Recent literature on the application of fractional derivative for studying the Covid‐19 virus includes Yao et al,^10^ with a study on fractional‐order Covid‐19 model with transmission route infected through the environment, while Baba et al^11^ employed the generalized Atangana‐Baleanu fractional derivative to investigate the awareness Covid‐19 epidemic model. A Fractional‐order Compartmental model of Covid‐19 vaccination with the fear factor was presented by Chatterjee et al.^12^ Ali et al^13^ presented a fractional‐order mathematical model for Covid‐19 outbreak with the effect of symptomatic and asymptomatic transmissions, and Baba et al^14^ assessed the efficiency of face‐mask to the community transmission of Covid‐19 using Fractional dynamical model. An Atangana‐Baleanu Caputo type fractional derivative model was used by Vijayalakshmi and Roselyn Besi^15^ to investigate the Covid‐19 with Self‐Protective Measures, and Ullah et al^16^ studied the control measures of overcoming Covid‐19 outbreaks via fractional‐order derivative model. By studying real data of omicron SARS‐CoV‐2 variant, Özköse et al^17^ investigated the fractional‐order modeling of the diseases with heart attack effect. A study by Omame et al^18^ investigated some Variants of Covid‐19 infection via Atangana‐Baleanu derivative, and Farman et al^19^ analyzed the outbreak of the Omicron Covid‐19 variant using the fractal‐fractional operator.

Following the above‐mentioned works, here, we simulate a fractional‐order mathematical model to describe the dynamics of the Covid‐19 epidemic. We divide the given research content into a number of sections, which is as follows: In Section 2, a complete model description in an integer‐order sense followed by a fractional‐order case is mentioned, where the main motivation to replace the integer‐order model with fractional‐order is to simulate the model dynamics more effectively because fractional‐order derivatives give more varieties in the simulations and the memory effects can only be described via fractional derivatives. In this regard, Section 3 is devoted to the stability analysis of the disease‐free and endemic equilibrium points. Complete practical simulations are explored in Section 4 where we adopted some parameter values based on the real data of Malaysia, where a number of numerical and graphical observations are justified. In the end, we came to a strong conclusion.

## 2 MODEL DESCRIPTION

Recently, Khan and Atangana^20^ studied the dynamic of the Covid‐19 pandemic and presented a mathematical model using a fractional derivative approach. The model provides a brief overview of the many types of interactions, the bat's first contact with their unknown hosts, which might likely be a wild animal. The other connection is between the interactions of individuals with the seafood at the market, which serves as a reservoir for the infection. As explained in the study, the primary cause of the infection is seafood when the unknown hosts and bats release the virus on seafood, which may include fish, toad, crayfish, and many more. As a result, when individuals purchase items already infected, they are more likely to become infected with the virus, either symptomatically or otherwise. The model was developed with the assumption that the seafood from the market had a high potential for infecting people who come to the market for transactions. In the following model (1), the author simplified the process by omitting the interacting ability between the bats and the hosts:

(1) $$\begin{array}{cc} \frac{dS_{p}}{dt} & =\Pi_{p}-\mu_{p}S_{p}-\frac{\eta_{p}S_{p}\left(\psi A_{p}+I_{p}\right)}{N_{p}}-\eta_{w}S_{p}M,\\ \frac{dE_{p}}{dt} & =\frac{\eta_{p}S_{p}\left(\psi A_{p}+I_{p}\right)}{N_{p}}+\eta_{w}S_{p}M-\left(1-\theta_{p}\right)w_{p}E_{p}-\theta_{p}\rho_{p}E_{p}-\mu_{p}E_{p},\\ \frac{dI_{p}}{dt} & =\left(1-\theta_{p}\right)w_{p}E_{p}-\left(\tau_{p}+\mu_{p}\right)I_{p},\\ \frac{dA_{p}}{dt} & =\theta_{p}\rho_{p}E_{p}-\left(\tau_{ap}+\mu_{p}\right)A_{p},\\ \frac{dR_{p}}{dt} & =\tau_{ap}A_{p}+\tau_{p}I_{p}-\mu_{p}R_{p},\\ \frac{dM}{dt} & =-\pi M+\varpi_{p}A_{p}+Q_{p}I_{p}. \end{array}$$

The following are the interpretations of various parameters considered in the model: The population of all the individuals is represented by N, and the susceptible is denoted by $S_{p}$; $E_{p}$ is defined to represent the exposed people. The symptomatic infected individuals are denoted by $I_{p}$ while, $A_{p}$ and $R_{p}$ represent asymptotic infected and the recovered/removed people, respectively. Also, the market is represented by M, the rate of birth is $\Pi_{p}$, and the coefficient for the virus transmission between septic and susceptible is denoted by $\eta_{p}$, while the natural death rate is given as $\mu_{p}$. The disease transmission coefficient from M to $S_{p}$ is represented by $\eta_{w}$. The multiple transmission of the asymptotic and asymptotic infected people is represented by ψ. Given ψ∈[0,1], this indicates that for ψ=0, the infection vanishes because there is no transmissible, while for ψ=1, a symptomatic‐like infection mechanism may likely occur. The fraction of the asymptomatic infection is represented by $\theta_{p}$. The rate of transmission for the infected individuals who have finished the incubation stage is $w_{p}$ and $\rho_{p}$, respectively. In addition, the removal or recovery rates for the symptomatic and asymptotically infected people are represented by $\tau_{p}$ and $\tau_{ap}$, respectively. $Q_{p}$ and $\varpi_{p}$ are the virus's contribution to the market by symptomatic and asymptomatic infected people, and π is the virus's removal rate from the market.

The focus of this study is on the fractional‐order model. The definition of fractional integral and the Caputo fractional derivative are given as follows:

Definition 1 The fractional integral of Reimann‐Liouville type of the fractional order $\beta \in {ℜ}^{t}$ of function x(t),t>0 is defined as

(2) $$I^{\beta }x(t)=\int_{0}^{t}\frac{{\left(t-s\right)}^{\beta -1}x(s)}{\Gamma (\beta )}\;ds,$$

where $t=t_{0}$ is the initial time and Γ(β) is the Euler's gamma function.

Definition 2 The Caputo fractional derivative (CFD) with order α∈n−1,n of function x(t),t>0 is defined as

(3) $${{cD}_{t}}^{\alpha }x(t)=I^{n-\alpha }D^{n}x(t),D_{t}=\frac{d}{dt}.$$

Using the above mentioned Caputo fractional derivatives of order 0<α≤1, we define the fractional‐order mathematical model as follows:

(4) $$\begin{array}{cc} {{cD}_{t}}^{\alpha }S_{p}(t)= & \;\Pi_{p}-\mu_{p}S_{p}-\frac{\eta_{p}S_{p}\left(\psi A_{p}+I_{p}\right)}{N_{p}}-\eta_{w}S_{p}M,\\ {{cD}_{t}}^{\alpha }E_{p}(t)= & \;\frac{\eta_{p}S_{p}\left(\psi A_{p}+I_{p}\right)}{N_{p}}+\eta_{w}S_{p}M-\left(1-\theta_{p}\right)w_{p}E_{p}-\theta_{p}\rho_{p}E_{p}-\mu_{p}E_{p},\\ {{cD}_{t}}^{\alpha }I_{p}(t)= & \;\left(1-\theta_{p}\right)w_{p}E_{p}-\left(\tau_{p}+\mu_{p}\right)I_{p},\\ {{cD}_{t}}^{\alpha }A_{p}(t)= & \;\theta_{p}\rho_{p}E_{p}-\left(\tau_{ap}+\mu_{p}\right)A_{p},\\ {{cD}_{t}}^{\alpha }R_{p}(t)= & \;\tau_{ap}A_{p}+\tau_{p}I_{p}-\mu_{p}R_{p},\\ {{cD}_{t}}^{\alpha }M(t)= & \;-\pi M+\varpi_{p}A_{p}+Q_{p}I_{p}, \end{array}$$

where 0<α≤1.

## 3 STABILITY ANALYSIS OF FRACTIONAL‐ORDER SYSTEM

This section considers the local stability analysis, based on the system of fractional‐order stability theory. It should be noted that while the equilibrium point of the fractional order is similar to the corresponding integer, its conditions are considerably different. When the eigenvalue is non‐negative, the equilibrium point for integer‐order is not stable but usually stable for fractional order.

Theorem 1 (Stability Analysis) The points of equilibrium for (6), where α∈(0,1] are said to be asymptotically (local) stable, if for the Jacobian matrix $\frac{\partial }{\partial y}f(t,y)$, all the eigenvalues $\lambda_{i}$ computed at the points of equilibrium satisfy $\left|\arg\;\lambda_{i}\right|>\frac{\alpha \pi }{2},\;i=1,2,3,4,5,6$. 

From the corresponding fraction‐order system given below,

(5) $${{cD}_{t}}^{\alpha }y_{i}(t)=f\left(t,y_{i}(t)\right),y_{i}\left(t_{o}\right)=y_{0},$$

where ${{cD}_{t}}^{\alpha }$ represents CFD of order α∈(0,1].

Next, the points of equilibrium would be evaluated using

(6) $${{cD}_{t}}^{\alpha }y_{i}(t)=0\Rightarrow f_{i}\left({f_{1}}^{eqn},{f_{2}}^{eqn},{f_{3}}^{eqn},{f_{4}}^{eqn},{f_{5}}^{eqn},{f_{6}}^{eqn}\right)=0,$$

for which we can get the equilibrium points ${f_{1}}^{eqn},{f_{2}}^{eqn},{f_{3}}^{eqn},{f_{4}}^{eqn},{f_{5}}^{eqn},{f_{6}}^{eqn}$. To compute for asymptotic stability, the system ${{cD}_{t}}^{\alpha }f(x)=f(x,y)$ would be considered in Caputo sense; let $y_{i}(t)={y_{i}}^{eqn}\epsilon_{i}(t)$. The following equilibrium points

$$\left({f_{1}}^{eqn},{f_{2}}^{eqn},{f_{3}}^{eqn},{f_{4}}^{eqn},{f_{5}}^{eqn},{f_{6}}^{eqn}\right)$$

are said to be locally asymptotically stable provided all the Jacobian eigenvalues

$$\left[\begin{array}{cccccc} \frac{\partial \left(f_{1}\right)}{\partial \left(y_{1}\right)} & \frac{\partial \left(f_{1}\right)}{\partial \left(y_{2}\right)} & \frac{\partial \left(f_{1}\right)}{\partial \left(y_{3}\right)} & \frac{\partial \left(f_{1}\right)}{\partial \left(y_{4}\right)} & \frac{\partial \left(f_{1}\right)}{\partial \left(y_{5}\right)} & \frac{\partial \left(f_{1}\right)}{\partial \left(y_{6}\right)}\\ \frac{\partial \left(f_{2}\right)}{\partial \left(y_{1}\right)} & \frac{\partial \left(f_{2}\right)}{\partial \left(y_{2}\right)} & \frac{\partial \left(f_{2}\right)}{\partial \left(y_{3}\right)} & \frac{\partial \left(f_{2}\right)}{\partial \left(y_{4}\right)} & \frac{\partial \left(f_{2}\right)}{\partial \left(y_{5}\right)} & \frac{\partial \left(f_{2}\right)}{\partial \left(y_{6}\right)}\\ \frac{\partial \left(f_{3}\right)}{\partial \left(y_{1}\right)} & \frac{\partial \left(f_{3}\right)}{\partial \left(y_{2}\right)} & \frac{\partial \left(f_{3}\right)}{\partial \left(y_{3}\right)} & \frac{\partial \left(f_{3}\right)}{\partial \left(y_{4}\right)} & \frac{\partial \left(f_{3}\right)}{\partial \left(y_{5}\right)} & \frac{\partial \left(f_{3}\right)}{\partial \left(y_{6}\right)}\\ \frac{\partial \left(f_{4}\right)}{\partial \left(y_{1}\right)} & \frac{\partial \left(f_{4}\right)}{\partial \left(y_{2}\right)} & \frac{\partial \left(f_{4}\right)}{\partial \left(y_{3}\right)} & \frac{\partial \left(f_{4}\right)}{\partial \left(y_{4}\right)} & \frac{\partial \left(f_{4}\right)}{\partial \left(y_{5}\right)} & \frac{\partial \left(f_{4}\right)}{\partial \left(y_{6}\right)}\\ \frac{\partial \left(f_{5}\right)}{\partial \left(y_{1}\right)} & \frac{\partial \left(f_{5}\right)}{\partial \left(y_{2}\right)} & \frac{\partial \left(f_{5}\right)}{\partial \left(y_{3}\right)} & \frac{\partial \left(f_{5}\right)}{\partial \left(y_{4}\right)} & \frac{\partial \left(f_{5}\right)}{\partial \left(y_{5}\right)} & \frac{\partial \left(f_{5}\right)}{\partial \left(y_{6}\right)}\\ \frac{\partial \left(f_{6}\right)}{\partial \left(y_{1}\right)} & \frac{\partial \left(f_{6}\right)}{\partial \left(y_{2}\right)} & \frac{\partial \left(f_{6}\right)}{\partial \left(y_{3}\right)} & \frac{\partial \left(f_{6}\right)}{\partial \left(y_{4}\right)} & \frac{\partial \left(f_{6}\right)}{\partial \left(y_{5}\right)} & \frac{\partial \left(f_{6}\right)}{\partial \left(y_{6}\right)} \end{array}\right]$$

computed at the points of equilibrium satisfy the condition $\left|\arg\;\lambda_{1,2,3,4,5,6}\right|>\frac{\alpha \pi }{2}$.^21^, ^22^, ^23^, ^24^ □

To derive the disease‐free and endemic stability and existence properties for the equilibrium points, we apply $R_{0}$, which represents an average of individual that can be infected by a patient.

If the above system (4) is equal to zero, two equilibria would be obtained, which include the point of equilibrium for the endemic denoted by $E^{e}$ and the equilibrium point for existence of disease‐free represented by $E^{0}$.

### 3.1 Disease‐free equilibrium, $E^{0}$

The disease‐free asymptotic stability $E^{0}$, for $R_{0}<1$, would be studied in this section. For the system (4), $R_{0}$, as defined by Khan and Atangana,^20^ is

$$R_{0}=\frac{\theta_{p}\rho_{p}\left(\mu_{p}+\tau_{p}\right)\left(\pi \psi \mu_{p}\eta_{p}+\varpi_{p}\Pi_{p}\eta_{w}\right)+\left(1-\theta_{p}\right)w_{p}\left(\tau_{ap}+\mu_{p}\right)\left(\Pi_{p}Q_{p}\eta_{w}+\pi \eta_{p}\mu_{p}\right)}{\pi \mu_{p}\left(\mu_{p}+\tau_{p}\right)\left(\tau_{ap}+\mu_{p}\right)\left(\theta_{p}\left(\rho_{p}-w_{p}\right)+\mu_{p}+w_{p}\right)}.$$

The disease‐free equilibrium is

(7) $$E^{0}=\left(S_{p}^{*}=\frac{\Pi_{p}}{\mu_{p}},E_{p}^{*}=0,I_{p}^{*}=0,A_{p}^{*}=0,R_{p}^{*}=0,M^{*}=0\right).$$

At $E^{0}$, then (4) is said be stable asymptotically if after computing for the eigenvalues of the Jacobian matrix via

(8) $$\left|\arg\;\lambda_{i}\right|>\frac{\alpha \pi }{2},$$

as presented in Theorem (1). This confirms the local stability of $E^{0}$ provided $R_{0}<1$; otherwise, when $R_{0}>1$, then it is unstable.

However, for the equilibrium of disease‐free $E_{E^{0}}$, the condition in (8) holds as discussed in Theorem 1.

Theorem 2 (Disease‐free equilibrium) The system (4) is said to be asymptotically locally stable at $E^{0}$ if and only if the following sufficient condition hold.

(9) $$R_{0}=\frac{\theta_{p}\rho_{p}\left(\mu_{p}+\tau_{p}\right)\left(\pi \psi \mu_{p}\eta_{p}+\varpi_{p}\Pi_{p}\eta_{w}\right)+\left(1-\theta_{p}\right)w_{p}\left(\tau_{ap}+\mu_{p}\right)\left(\Pi_{p}Q_{p}\eta_{w}+\pi \eta_{p}\mu_{p}\right)}{\pi \mu_{p}\left(\mu_{p}+\tau_{p}\right)\left(\tau_{ap}+\mu_{p}\right)\left(\theta_{p}\left(\rho_{p}-w_{p}\right)+\mu_{p}+w_{p}\right)}<1.$$

The proof of Theorem 2 follows from results of Jacobian (4). If we can obtain a negative real root at $E^{0}$ for all the eigenvalues of (4), then this prove of the theorem is complete. Therefore, we get

$$\left[\begin{array}{cccccc} -\mu_{p} & 0 & -\frac{\Pi_{p}\eta_{p}}{\mu_{p}{Nu}_{p}} & 0 & 0 & -\frac{\eta_{w}\Pi_{p}}{\mu_{p}}\\ 0 & -\left(1-\theta_{p}\right)w_{p}-\theta_{p}\rho_{p}-\mu_{p} & \frac{\Pi_{p}\eta_{p}}{\mu_{p}{Nu}_{p}} & 0 & 0 & \frac{\eta_{w}\Pi_{p}}{\mu_{p}}\\ 0 & \left(1-\theta_{p}\right)w_{p} & -\tau_{p}-\mu_{p} & 0 & 0 & 0\\ 0 & \theta_{p}\rho_{p} & 0 & 0 & 0 & 0\\ 0 & 0 & \tau_{p} & 0 & -\mu_{p} & 0\\ 0 & 0 & Q_{p} & 0 & 0 & -\mu_{p} \end{array}\right]$$

Then, for

$$S_{eqn},E_{eqn},I_{eqn},A_{eqn},R_{eqn},M_{eqn}=\left(S_{p}^{*}=\frac{\Pi_{p}}{\mu_{p}},E_{p}^{*}=0,I_{p}^{*}=0,A_{p}^{*}=0,R_{p}^{*}=0,M^{*}=0\right),$$

we have

$$A=\left[\begin{array}{cccccc} -\mu_{p} & 0 & -\frac{\eta_{p}\Pi_{p}}{\mu_{p}{Nu}_{p}} & 0 & 0 & -\frac{\eta_{w}\Pi_{p}}{\mu_{p}}\\ 0 & -\left(1-\theta_{p}\right)w_{p}-\theta_{p}\rho_{p}-\mu_{p} & \frac{\eta_{p}\Pi_{p}}{\mu_{p}{Nu}_{p}} & 0 & 0 & \frac{\eta_{w}\Pi_{p}}{\mu_{p}}\\ 0 & \left(1-\theta_{p}\right)w_{p} & -\tau_{p}-\mu_{p} & 0 & 0 & 0\\ 0 & \theta_{p}\rho_{p} & 0 & 0 & 0 & 0\\ 0 & 0 & \tau_{p} & 0 & -\mu_{p} & 0\\ 0 & 0 & Q_{p} & 0 & 0 & -\mu \end{array}\right],$$

and its eigenvalues

(10) $$\begin{array}{cc} & [-\mu_{p}],\\ & [-\mu_{p}]. \end{array}$$

It is obvious that (10) is less than zero, implying that $R_{0}<0$ and the condition in (8) are satisfied. As a result, the eigenvalues of the system (4) are always negative (because of the positive parameters). Thus, (4) is locally asymptotically stable. The equilibrium of the disease‐free E0 is locally asymptotically stable. On the other hand, it will be unstable if

(11) $$R_{0}=\frac{\theta_{p}\rho_{p}\left(\mu_{p}+\tau_{p}\right)\left(\pi \psi \mu_{p}\eta_{p}+\varpi_{p}\Pi_{p}\eta_{w}\right)+\left(1-\theta_{p}\right)w_{p}\left(\tau_{ap}+\mu_{p}\right)\left(\Pi_{p}Q_{p}\eta_{w}+\pi \eta_{p}\mu_{p}\right)}{\pi \mu_{p}\left(\mu_{p}+\tau_{p}\right)\left(\tau_{ap}+\mu_{p}\right)\left(\theta_{p}\left(\rho_{p}-w_{p}\right)+\mu_{p}+w_{p}\right)}>1.$$

□

### 3.2 Endemic equilibrium, $E^{e}$

Based on (4), the endemic points, $E^{e}$, can be obtained if we solve the quadratic equation, $\lambda^{6}+A\;\lambda^{5}+B\;\lambda^{4}+C\;\lambda^{3}+D\;\lambda^{2}+E$. We define $E^{e}=(S_{p}^{*},E_{p}^{*},I_{p}^{*},A_{p}^{*},R_{p}^{*},M^{*})$ as the point of endemic for (4). Other results on equilibrium of the endemic will follow in the subsequent section.

## 4 EXPERIMENTAL SIMULATIONS

A multi‐step numerical scheme known as the Adams Predictor‐Corrector method was employed for all the simulations of this study. The Adams‐Bashforth method, which was first considered in Diethelm et al,^25^ uses the solutions in previous instants to explicitly compute the approximate solution at an instant time. Considering the previous information would increase the accuracy of the results. The method was further studied by El‐Saka^21^ and Ameen and Novati,^26^ to possess an error‐free approach for obtaining the solution of a problem with a logical and sensible choice of time step.^26^ The Adams‐type Predictor‐Corrector approach can further be considered to solve other numerical problems such as nonlinear differential equations^27^ and Fractional Shimizu–Morioka problems.^28^

To demonstrate the model stability as considered in (4), the value of parameters are taken as follows: The initial values are $S_{p}(0)=32351818;E_{p}(0)=31927442;I_{p}(0)=389846;A_{p}(0):=200;R_{p}(0):=389846;M(0):=50000,\;\theta_{p}=0.413,\;\eta_{w}=0.000001231,\;\mu_{p}=0.00500,\;\eta_{p}=0.05,\;\Pi_{p}=107644.22451,\;w_{p}=0.00047876,\rho_{p}=0.005,\tau_{p}=0.09871,\;\tau_{ap}=0.3912,\;Q_{p}=0.000298,\;\varpi_{p}=0.0001,\;\pi =0.01$.

The computation of equilibrium points for the model (4) is given below:

$$E_{1}(S_{p_{1}},E_{p_{1}},I_{p_{1}},A_{p_{1}},R_{p_{1}},M_{1})=(0.99,0,0,0,0,0),$$

and

$$\begin{array}{cc} E_{2}(S_{p_{2}},E_{p_{2}},I_{p_{2}},A_{p_{2}},R_{p_{2}},M_{2})= & \;(2.379283090*10^{8},-1.957934579*10^{8},-4.478254622*10^{5},\\ & -3.338181529*10^{5},-4.135310852*10^{7},-5752.393507). \end{array}$$

Thus, the Jacobian of the corresponding equilibrium point $\left(R_{p1},I_{p1}\right)$ is as follows:

$$J=\left[\begin{array}{cccccc} -H^{*} & 0 & -0.000000003050994878\;S_{p} & 0 & 0 & -0.0000015\;S_{p}\\ H^{*}* & -0.012152 & 0.000000003050994878\;S_{p} & 0 & 0 & 0.0000015\;S_{p}\\ 0 & 0.000430 & -0.188 & 0 & 0 & 0\\ 0 & 0.001722 & 0 & 0 & 0 & 0\\ 0 & 0 & 0.178 & 0 & -0.01 & 0\\ 0 & 0 & 0.000398 & 0 & 0 & -0.06 \end{array}\right]$$

where

$$\begin{array}{cc} H^{*}= & \;0.01-1.525497439\times 10^{-11}\;A_{p}-0.000000003050994878\;I_{p}-0.0000015\\ M= & \;1.303^{-2}+1.210^{-10}\;A_{p}+6.049^{-9}\;I_{p}+1.231^{-6}\;M,\\ H^{*}*= & \;1.525497439\times 10^{-11}\;A_{p}+0.000000003050994878\;I_{p}+0.0000015\;M, \end{array}$$

and the disease‐free $E^{0}$ eigenvalues are

$$\lambda_{i}=\left[\begin{array}{c} -0.0100000000000000+0.0\;I_{p}\\ -0.187999999609502+0.0\;I_{p}\\ 8.631409473\times 10^{-18}+0.0\;I_{p}\\ -0.0121519962409175+0.0\;I_{p}\\ -0.0100000000000000+0.0\;I_{p}\\ -0.0600000041495804+0.0\;I_{p} \end{array}\right];$$

that of the endemic, $E^{e}$ is

$$\lambda_{i}=\left[\begin{array}{c} -0.187088526906394+0.0\;I_{p}\\ 0.00564108167009003+0.0\;I_{p}\\ 8.346796282\times 10^{-17}+0.0\;I_{p}\\ -0.0112025181090758+0.0\;I_{p}\\ -0.0100000000000001+0.0\;I_{p}\\ -0.0675020408166201+0.0\;I_{p} \end{array}\right],$$

while the pandemic models' characteristic equation as presented in (4) is

$$\begin{array}{cc} P= & \;\lambda^{6}+0.2826921507\;\lambda^{5}+0.02028154803\;\lambda^{4}+0.0004914900591\;\lambda^{3}\\ & +0.000004874756376\;\lambda^{2}+0.00000001715318437\;\lambda . \end{array}$$

The argument $\left|\arg\;\lambda_{1,2,3,4,5,6}\right|$ of the Jacobian J at α=0.8 falls within the value range 3.141592654. These values for $\left|\arg\;\lambda_{1}\right|$ for the points $\left(S_{p},E_{p},I_{p},A_{p},R_{p},M\right)$ are stable, and the system possesses the asymptotic stability as a result of eigenvalues fulfilling $\left|\arg\;\lambda_{1}\right|>\frac{\alpha \pi }{2}$. This implies $\left|\arg\;\lambda_{1}\right|=3.141592654>2.000000000=\frac{\alpha \pi }{2}$.

Also, it is obvious that using direct calculation,

$$R_{0}=\frac{\theta_{p}\rho_{p}\left(\mu_{p}+\tau_{p}\right)\left(\pi \psi \mu_{p}\eta_{p}+\varpi_{p}\Pi_{p}\eta_{w}\right)+\left(1-\theta_{p}\right)w_{p}\left(\tau_{ap}+\mu_{p}\right)\left(\Pi_{p}Q_{p}\eta_{w}+\pi \eta_{p}\mu_{p}\right)}{\pi \mu_{p}\left(\mu_{p}+\tau_{p}\right)\left(\tau_{ap}+\mu_{p}\right)\left(\theta_{p}\left(\rho_{p}-w_{p}\right)+\mu_{p}+w_{p}\right)}$$

is equal to 0.01421842382, which are compatible and in excellent agreement with Theorem 2 (disease‐free equilibrium), with $R_{0}<0.0.01421842382$. This means that the above‐mentioned conditions for asymptotic stability and existence are satisfied. It also follows that the transmission of a disease is determined by the number of people who come in contact with an infected person in the population. The model's behavior is also influenced by the basic reproduction number, $R_{0}$, which is the average number of persons infected by one sick person. The existence and stability conditions at the equilibrium points were established using $R_{0}$. When $R_{0}>1$, a standard infective cause on average above one secondary infection, leading to a pandemic, this value establishes a threshold for pandemic processes. Otherwise, with $R_{0}<1$, infective agents on average often result in less than one secondary infection, therefore infection prevalence cannot rise in this situation.

Now, for the purpose of novelty in the graphical simulations, we replace the above old parameter values with some new parameter values, which are simulated with the help of real data of Covid‐19 in Malaysia. The data are collected between November 2020 to April 2021. The values of parameters are taken as follows: The initial values are $S_{p}(0)=32351818;E_{p}(0)=31927442;I_{p}(0)=389846;A_{p}(0):=200;R_{p}(0):=389846;M(0):=50000,\;\theta_{p}=0.413,\;\eta_{w}=0.000001231,\;\mu_{p}=0.00500,\;\eta_{p}=0.05,\;\Pi_{p}=107644.22451,\;w_{p}=0.00047876,\rho_{p}=0.005,\tau_{p}=0.09871,\;\tau_{ap}=0.3912,\;Q_{p}=0.000298,\;\varpi_{p}=0.0001,\;\pi =0.01$.

**FIGURE 1:**
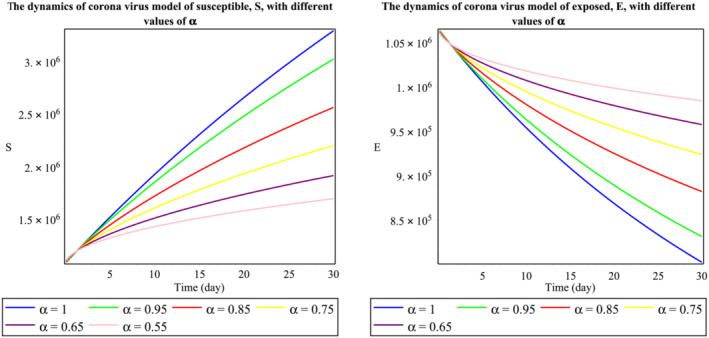
Behavior of S and E classes respect to time t (daily) [Colour figure can be viewed at wileyonlinelibrary.com]

**FIGURE 2:**
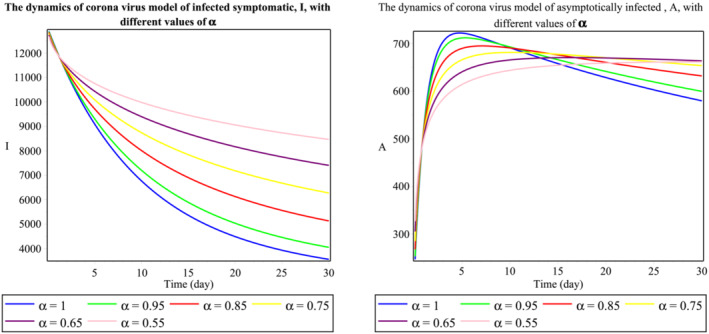
Behavior of I and A classes respect to time t (daily) [Colour figure can be viewed at wileyonlinelibrary.com]

**FIGURE 3:**
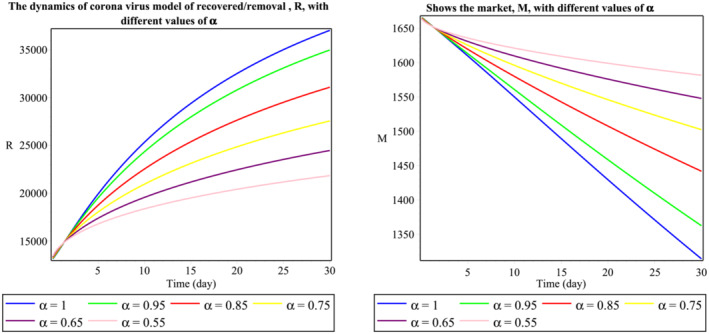
Behavior of R and M classes respect to time t (daily) [Colour figure can be viewed at wileyonlinelibrary.com]

The group of Figure 1 shows the dynamics of the second wave of the daily reported cases of 2019‐nCoV in Malaysia. It indicates the number of individuals in a certain time, t (days), and stable endemic equilibrium. Here, we see that an increase in the fractional‐order values will decrease the population of exposed individuals sharply. From Figure 2, we notice that increment in the fractional‐order decreases the population of the symptomatic infected population but provides some random changes in the asymptomatic infected population. Similarly, the changes in the recovered class are observed in Figure 3. Therefore, we can see that the fractional‐order simulations are much stronger as compared to integer‐order observations because of more degree of freedom in the graphical observations. Findings from this study are compared to those obtained using Atangana‐Baleanu derivative approach, which was simulated in the source study.^20^

## 5 CONCLUSION

The coronavirus (COVID‐19) pandemic model was considered in this work. The Caputo derivative has been used to define fractional ordinary differential equations. To obtain an approximation to the solution of the fractional‐order model, an Adams‐type predictor‐corrector approach with α∈(0,1] is employed. Results from the study show that the behavior of the model is affected by the basic reproduction number $R_{0}$. Also, $R_{0}$ was applied to establish the stability and existence conditions at the points of equilibrium. Using novel parameter values based on Malaysian data makes this study more visible to the literature. Results from this study show that this new approach is very effective and can be studied as an alternate method for solving fractional differential problems having similar dynamics. This study will also be important to medical authorities for predicting the future dynamics of Covid‐19.

## CONFLICT OF INTEREST

The authors declare no conflict of interest. Also, this study is not funded nor supported by any grant.
